# Shaping the Future of Obesity Treatment: In Silico Multi-Modeling of IP6K1 Inhibitors for Obesity and Metabolic Dysfunction

**DOI:** 10.3390/ph17020263

**Published:** 2024-02-19

**Authors:** Ismail Mondal, Amit Kumar Halder, Nirupam Pattanayak, Sudip Kumar Mandal, Maria Natalia D. S. Cordeiro

**Affiliations:** 1 Dr. B. C. Roy College of Pharmacy and Allied Health Sciences, Dr. Meghnad Saha Sarani, Bidhannagar, Durgapur 713206, India; ismail.mondal@bcrcp.org (I.M.); amit.halder@fc.up.pt (A.K.H.); nirupam.chottu@gmail.com (N.P.); sudip.mandal@bcrcp.org (S.K.M.); 2 LAQV@REQUIMTE, Department of Chemistry and Biochemistry, Faculty of Sciences, University of Porto, 4169-007 Porto, Portugal

**Keywords:** IP6K1 inhibitors, QSAR, pharmacophore mapping, homology modeling, molecular dynamics simulations

## Abstract

Recent research has uncovered a promising approach to addressing the growing global health concern of obesity and related disorders. The inhibition of inositol hexakisphosphate kinase 1 (IP6K1) has emerged as a potential therapeutic strategy. This study employs multiple ligand-based in silico modeling techniques to investigate the structural requirements for benzisoxazole derivatives as IP6K1 inhibitors. Firstly, we developed linear 2D Quantitative Structure–Activity Relationship (2D-QSAR) models to ensure both their mechanistic interpretability and predictive accuracy. Then, ligand-based pharmacophore modeling was performed to identify the essential features responsible for the compounds’ high activity. To gain insights into the 3D requirements for enhanced potency against the IP6K1 enzyme, we employed multiple alignment techniques to set up 3D-QSAR models. Given the absence of an available X-ray crystal structure for IP6K1, a reliable homology model for the enzyme was developed and structurally validated in order to perform structure-based analyses on the selected dataset compounds. Finally, molecular dynamic simulations, using the docked poses of these compounds, provided further insights. Our findings consistently supported the mechanistic interpretations derived from both ligand-based and structure-based analyses. This study offers valuable guidance on the design of novel IP6K1 inhibitors. Importantly, our work exclusively relies on non-commercial software packages, ensuring accessibility for reproducing the reported models.

## 1. Introduction

Over the past four decades, the global prevalence of obesity has surged unrelieved, transcending age, race, and gender boundaries [1,2]. This alarming trend has led to a cascade of health issues, including type 2 diabetes mellitus (T2DM), hypertension, dyslipidemia, cardiovascular diseases, non-alcoholic fatty liver disease/non-alcoholic steatohepatitis (NAFLD/NASH), reproductive dysfunction, respiratory abnormalities, psychiatric and neurodegenerative conditions, and even specific malignancies [3,4,5]. Fortunately, a combination of medication and lifestyle modifications has demonstrated positive effects in the battle against obesity [6,7]. Remarkably, even a modest 5–10% reduction in body weight or fat can significantly lower the risk of obesity-related disorders in adults [8,9]. Yet, achieving sustained weight loss remains a challenge, prompting intensive research into novel therapeutic strategies to combat obesity and its associated metabolic ills [2,10]. 

A family of enzymes known as inositol hexakisphosphate kinases (IP6Ks) plays a pivotal role in phosphorylating inositol hexakisphosphate (InsP6) to yield 5-diphosphoinositol pentakisphosphate (5-InsP7 or 5PP-IP5, also abbreviated as IP7). This phosphorylation marks the critical inaugural step in the synthesis of inositol pyrophosphates (PP-InsPs). Recent studies have revealed the potential of the PP-InsP biosynthesis pathway as a therapeutic target for metabolic disorders, osteoporosis, thromboembolism, infection, cancer metastasis, and aging-related conditions [11,12]. Inositol pyrophosphates, in essence, serve as highly energetic eukaryotic messenger molecules that underpin essential physiological processes, including ATP generation, insulin secretion, cell signaling, cell migration, DNA repair, and the maintenance of bioenergetic balance [13,14,15]. 

Among the three primary IP6K isoforms, IP6K1 and IP6K2 are ubiquitously expressed in almost all tissues, while IP6K3 is confined to the heart, skeletal muscle, and brain [16]. IP6K1 exerts a dual effect by inhibiting certain elements of insulin signaling and promoting insulin secretion from the pancreatic cells. Moreover, it curtails adipocyte thermogenesis, leading to a decrease in the total energy expenditure in the body [17]. Interestingly, IP6K1 knockout in mice results in improved insulin sensitivity and energy expenditure, providing protection against diet-induced obesity, hyperinsulinemia, and insulin resistance. Reduced IP6K1 levels also prove beneficial in the treatment of NAFLD and NASH [18]. These findings underscore the potential of developing IP6K1 inhibitors as therapeutic candidates to combat obesity and related metabolic disorders [18,19,20,21].

Heterocyclic compounds are commonly employed in medicinal chemistry research for the design and development of novel lead compounds aimed at promising therapeutic targets [22,23,24,25]. In a recent breakthrough, Zhou et al. unveiled a series of benzisoxazole derivatives displaying varying degrees of inhibitory potential against the IP6K1, IP6K2, and IP6K3 enzymes [26]. In vitro profiling of the HCT116 colon cancer cell line highlighted that potent inhibitors of IP6K1 and IP6K2 can significantly reduce the inositol pyrophosphate levels without affecting other inositol phosphates. Notably, one of these potent inhibitors lowered the inositol pyrophosphate levels by 66–81% without majorly perturbing any other inositol phosphates. Furthermore, in vivo studies demonstrated that these derivatives could alleviate obesity-related pathological complications and reduce body weight without affecting food intake. 

Computer-aided drug design is currently considered one of the most crucial approaches in pre-clinical drug discovery [27,28,29,30,31]. In the present study, we embarked on a comprehensive in silico modeling effort with this significant series of compounds to unravel the structural prerequisites for their enhanced inhibitory potential against IP6K1. Intriguingly, a high correlation (*R*^2^~0.85) emerged between the activities of these compounds against IP6K1 and IP6K2, suggesting that the structural attributes identified in IP6K1 inhibition are broadly applicable. We employed 2D Quantitative Structure–Activity Relationship (2D-QSAR) and 3D-QSAR modeling and ligand-based pharmacophore mapping to disclose these attributes. To bolster our findings, we compared the insights derived from these ligand-based in silico modeling analyses with the results of molecular dynamics (MD) simulations using a homology model of IP6K1. Our investigation not only contributes to the design of novel IP6K1 inhibitors but also holds promise in the quest to combat obesity.

## 2. Results and Discussions 

### 2.1. The 2D-QSAR Modeling

Following the outlined strategy described in Materials and Methods, we began by seeking the best linear models relating the inhibitory activity and the calculated alvaDesc descriptors using both the SFS and GA feature selection algorithms. Initially, the 2D-QSAR MLR models were built using selected alvaDesc descriptors known for their ease of interpretation. Subsequently, we incorporated all descriptors to assess potential improvements in the model’s predictivity. A comprehensive summary of the results is shown in Table 1 for the models (M01–M09) developed with interpretable descriptors and the models (M10–M18) incorporating all descriptors.

As can be seen, the GA algorithm yielded the most predictive interpretable MLR model, while the SFS algorithm with the NMAE scoring function and five-fold cross-validation provided the most statistically significant MLR model using all descriptors. Comparison between the models M09 (developed with interpretable descriptors) and M15 (utilizing all descriptors) revealed a significant improvement in predictivity when all the descriptors were employed. Despite this, the statistical predictivity of model M09 remained satisfactory, especially considering the limited number of features used in its development. Additionally, we observed that models M10–M18 are notably rich in 2D and 3D topological descriptors. Therefore, it is important to consider both the M09 and M15 models, as the former helps us understand the structural requirement whereas the latter provides a better predictive model, highlighting the descriptors that more accurately represent the structural requirements. The detailed statistical results for models M09 and M15 are presented in Table 2. 

It is noteworthy that we initially included five descriptors for the model development considering the presence of 29 compounds in the training set, which satisfies the criterion of a 1:5 ratio between the number of independent variables and the total number of training set data points. However, to assess whether a lower number of descriptors was sufficient for producing a statistically reliable model, we employed a 5% increment strategy using the SFS-QSAR-tool_v2. In this strategy, a descriptor is added sequentially to the model only if it improves the *Q*^2^_LOO_ of the model by at least 5%. For both M09 and M15, five descriptors were selected, indicating that five descriptors are indeed required in these models. 

**Figure 1:**
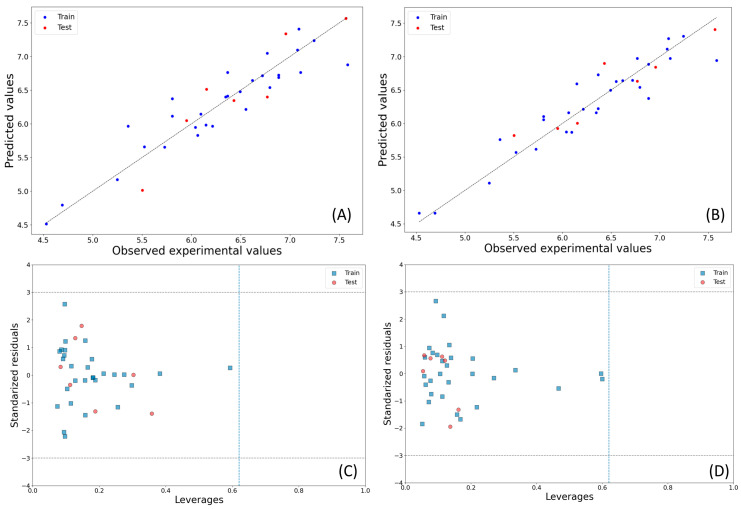
Observed vs. predicted activity plots for models M09 (**A**) and M15 (**B**), along with their corresponding Williams plots: M09 (**C**) and M15 (**D**).

The observed vs. predicted activity plots for models M09 and M15 are shown in Figure 1. Beyond the *Q*^2^_LOO_ and *R*^2^_Pred_ values, the other internal and external validation parameters were found to be satisfactory for both models (for example, MAE: 0.201 for M09, 0.181 for M15). Additionally, the conditions critical for linear regression model acceptance were also met; for example, no high inter-collinearity was observed among the descriptors (maximum *R*: 0.469 and 0.404). Multicollinearity was assessed using the VIF values, all of which remained below 2.0, indicating its absence. Furthermore, the c*R*_p_^2^ values for M09 and M15 were 0.761 and 0.794, respectively, confirming the uniqueness and non-random development of both models. 

**Figure 2:**
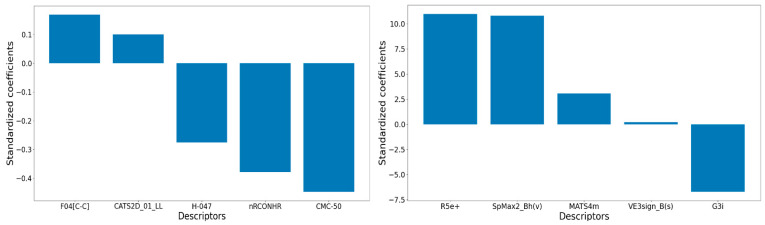
Relative significance of the descriptors of models M09 (**left**) and M15 (**right**).

Figure 1 also displays the Williams plots of the best 2D-QSAR models found, offering an assessment of their applicability domain. Importantly, no structural or response outliers are identified in both these models. Figure 2 shows the relative significance of their descriptors according to their standardized coefficients.

**Figure 3:**
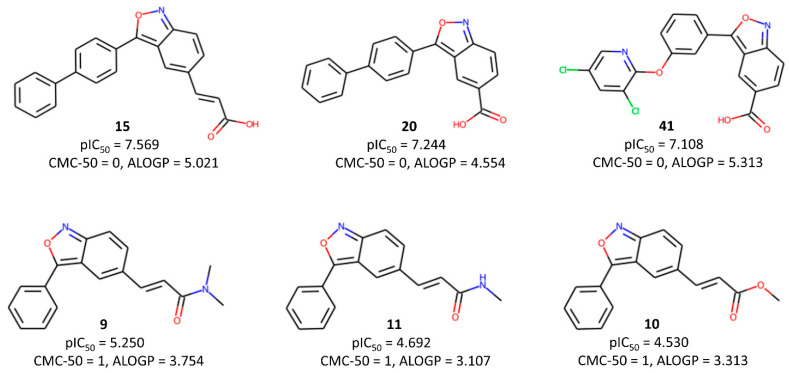
Typical examples highlighting the importance of the CMC-50 descriptor to the inhibitory activity.

Let us initially focus on discussing the descriptors that are part of model M09. Its most significant descriptor is CMC-50, a drug-like index representing the Ghose–Viswanadhan–Wendoloski CMC (Comprehensive Medicinal Chemistry) drug-like index at 50%. A negative correlation between this descriptor and pIC_50_ indicates that lower values of CMC-50 are favorable for higher biological activity. CMC-50 is basically a binary descriptor that depends on the values of the following quantities: ALOGP (Ghose–Crippen octanol–water partition coefficient), AMR (Ghose–Crippen molar refractivity), MW (molecular weight), and nAT (number of atoms). Compounds with an ALOGP between 1.3 and 4.1, an AMR between 70 and 110, an MW between 230 and 390, and an nAT between 30 and 55 receive a value of 1; otherwise, it is 0 (see Figure 3). A careful analysis reveals that most of the higher-activity compounds indeed possess a low value for this descriptor. Above all, a higher lipophilicity (ALOGP) seems to be the key factor influencing this descriptor, suggesting that hydrophobic interactions play a significant role in determining the biological activity of these compounds.

**Figure 4:**
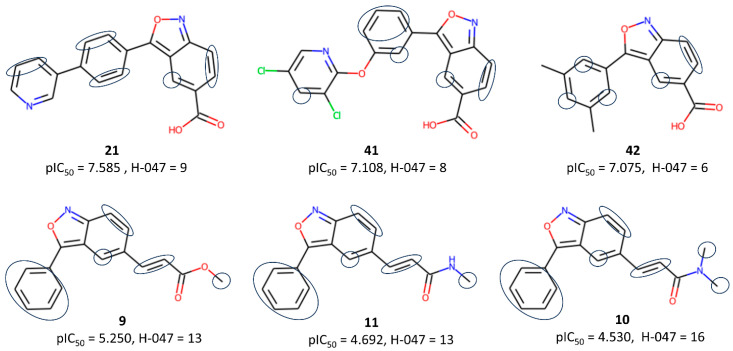
Typical examples highlighting the importance of the H-047 descriptor to the inhibitory activity.

The second most influential descriptor of model M09 is the functional group count descriptor nRCONHR, which simply stands for the number of secondary amides (aliphatic). Its associated negative coefficient implies that a larger number of lower-activity compounds are part of this functional group. The third crucial descriptor, H-047 (hydrogen atom attached to C1(sp^3^)/C0(sp^2^), in which C1 stands for a sp^3^-hybridized carbon that is not attached to one heteroatom, whereas C0 represents a sp^2^-hybridized carbon that is not attached to any heteroatom, shows higher values in low-activity compounds (see Figure 4). The presence of amide and ester side chains or unsubstituted phenyl rings in low-activity compounds is mainly responsible for the higher value of this descriptor and the observed low activity.

**Figure 5:**
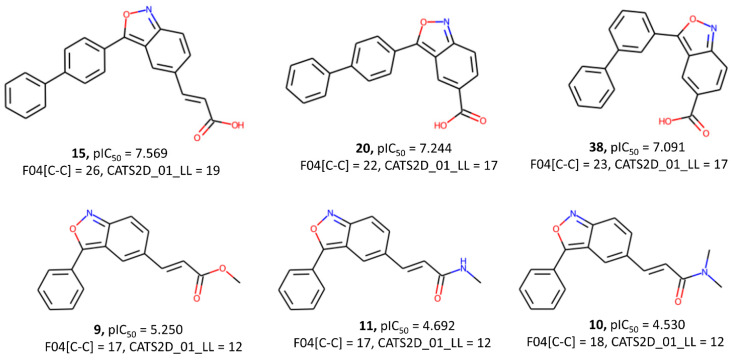
Typical examples highlighting the importance of both descriptors, F04[C-C] and CATS2D_01_LL, to the inhibitory activity.

Additionally, the F04[C-C] descriptor, representing the frequency of two carbon atoms located at a topological distance of 4, is associated with increased biological activity. Chemically Advanced Template Search (CATS) descriptors are a very important group of descriptors that count the pharmacophore features (2D or 3D) within the topological distance between two such features. For example, CATS2D_01_LL in M09 counts the total number of instances in a molecule where two lipophilic features are separated by a topological distance of 1. The positive correlation of both F04[C-C] and CATS2D_01_LL with the response variable emphasizes the crucial role of hydrophobic interactions in determining a higher affinity to the IP6K1 receptor (see Figure 5).

Moving on to a discussion of the descriptors in model M15, Table 3 offers details on the meaning of these descriptors. It is worth noting here that all the descriptors in this model are complex graph-based topological descriptors. The descriptors R5e+ and G3i belong to the category of 3D descriptors, and their values depend on the specific 3D conformation of the compounds. The remaining are 2D descriptors. Given the highly satisfactory statistical predictivity obtained from this model, this implies that the specific topology of the compounds plays a crucial role in determining the biological activity of these compounds. However, the two most influential descriptors in the model, R5e+ and SpMax2_Bh(v), have almost the same relative significance. These two descriptors are weighted by electronegativity and van der Waals volume, respectively. Therefore, this suggests that, along with the 3D geometry of the compounds, the electrostatic and hydrophobic interactions of the compounds are involved in higher biological activity. The third most significant descriptor of the model is associated with the ionization potential, which is related to the polarity of the compounds, whereas MATS4m is associated with the atomic mass. Overall, M09 mainly highlights the importance of hydrophobicity for higher activity, whereas the descriptors of M15 indicate that a balance between hydrophobic/steric and electrostatic interactions is required for higher activity.

### 2.2. Ligand-Based Pharmacophore Mapping

After identifying the structural requirements using 2D-QSAR modeling, ligand-based pharmacophore mapping was employed to develop predictive models and understand the pharmacophore features associated with improved biological potency against IP6K1. The QPHAR tool was utilized to generate the ligand-based pharmacophore models, dividing the dataset into 70% (training) and 30% (test) sets. The conformers generated using the genetic algorithm yielded better models than those created using Confab. The results of the most predictive pharmacophore model are presented in Table 4.

The model demonstrated a satisfactory internal predictivity with an *R*^2^ of 0.845 and RMSE of 0.309. For external validation, *R*^2^_Pred_ was found to be greater than the cut-off value of 0.50. However, removing one compound improved *R*^2^_Pred_ to 0.716. It is important to note here that QPHAR selects the most rigid structure of the dataset as the template structure. 

**Figure 6:**
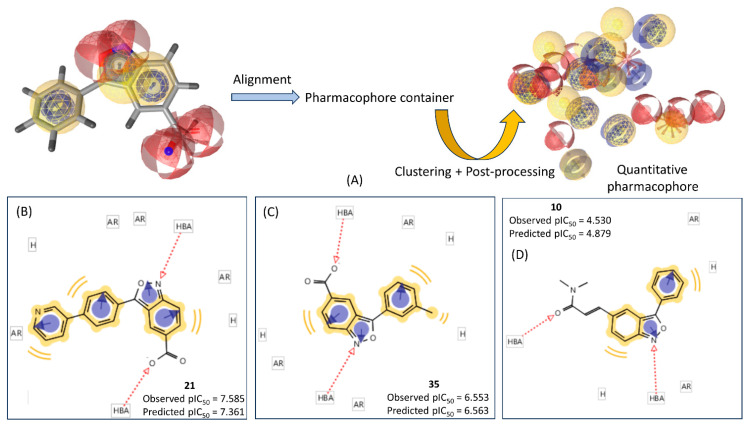
(**A**) Pharmacophore features of the template molecule alongside the generated quantitative pharmacophore. The pharmacophore-aligned structures of compounds **21** (**B**), **35** (**C**), and **10** (**D**), along with the pharmacophore features, are also displayed.

The template pharmacophore aligned with compound **17** is shown in Figure 6, along with the pharmacophore container and the final quantitative pharmacophore (i.e., hpmodel). Additionally, the pharmacophore alignments of three compounds (**21**, **35**, and **10**) with different biological activities are displayed.

Among these three compounds, **21** is the most potent (pIC_50_ = 7.585), **10** is the least active (pIC_50_ = 4.530), and **35** is a compound with intermediate potency (pIC_50_ = 6.553). Noticeably, the potencies of these compounds against IP6K1 were accurately predicted using the QPHAR model. The hydrophobic and aromatic ring features of these compounds decreased steadily with a decreasing affinity while the number of hydrogen bond acceptor features remained constant at two for each compound. These results align with the findings of the 2D-QSAR analyses, suggesting that hydrophobic interactions may play crucial roles in ligand binding to the IP6K1 receptor. However, the comprehensive QSAR model (M15), which considered both 2D and 3D descriptors, hinted that polar interactions may also be crucial. Specifically, in compound **21**, four aromatic ring (AR) features are observed, while **35** and **10** have three and two AR features, respectively. Both **21** and **10** are aligned with two hydrophobic features, whereas **35** is aligned with three hydrophobic features. Comparing **35** and **10**, the former contains two additional major features, i.e., one aromatic ring and one hydrophobic feature. These differences in features may explain the variation in the biological activity between these two compounds.

### 2.3. The 3D-QSAR Analysis

To gain deeper insights into the structural requirements for potency against IP6K1, we performed a 3D-QSAR analysis using the open-source Open3DQSAR software and following the alignment techniques and feature selection strategies previously mentioned. The utilization of the rigid body alignment technique yielded more robust and predictive statistical results, as illustrated in Table 5. The alignment process, crucial for the development of these models, is visually depicted in Figure 6.

As can be observed, the FFD-SEL technique is proven to be particularly effective, yielding highly satisfactory internal and external predictivity with *Q*^2^_LOO_ and *R*^2^_Pred_ values of 0.637 and 0.747, respectively. Given the high sensitivity of 3D-QSAR models, especially in the context of small datasets, the model is demonstrated to have a consistently moderate to satisfactory level of predictivity. What sets this model apart is its intrinsic uniqueness, evident in the substantial deterioration of cross-validation results upon scrambling the response variable. 

**Figure 7:**
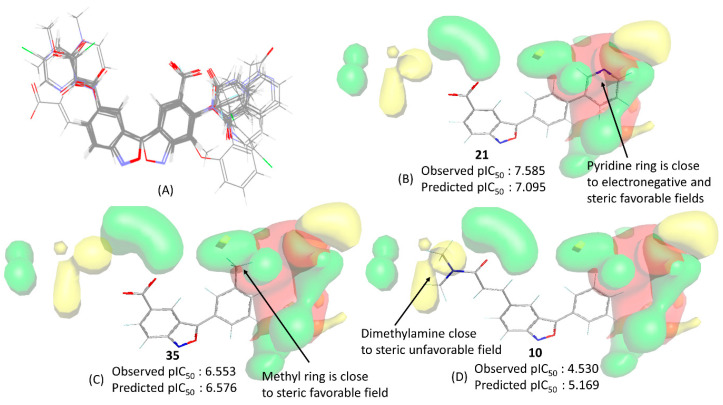
(**A**) Aligned structures for the dataset compounds. Contour maps obtained using the best 3D-QSAR model for compounds (**B**) **21**, (**C**) **35**, and (**D**) **10**. The color codes used range from green (steric favorable) to red (electrostatic favorable).

Figure 7 showcases the contour maps generated using the FFD-SEL technique, featuring compounds **25**, **31**, and **10**. These visual representations offer a comprehensive view of the steric and electrostatic contributions, with steric and electrostatic elements accounting for 60% and 40%, respectively. The contours delineate two predominant regions: on the right side, steric and electrostatic contours coexist, while the left side exclusively features steric contours. The significance of the steric and electrostatic contours on the right becomes evident in their crucial roles in determining a higher potency.

Take, for example, the most potent compound, **21**, where favorable steric and electronegative interactions near its pyridine moiety suggest its involvement in both hydrophobic and electrostatic interactions with the receptor. In contrast, compound **35**, with intermediate activity, does not fully engage with the electronegative favorable contour but shows improved activity due to the insertion of its methyl group into the hydrophobic favorable contour. Conversely, the least active compound, **10**, completely avoids these contours, with its dimethylamine moiety in the side chain closely aligning with the steric unfavorable contour. Remarkable also is that no contour map is found near the benzisoxazole ring of these compounds, indicating effective superimposition according to rigid body alignment. 

### 2.4. Homology Modeling of IP6K1 and MD Simulations

To date, no X-ray crystallographic structure of IP6K1 has been disclosed. Therefore, we need to rely on its homology model for structure-based modeling. In this work, we performed homology modeling using UniProt ID Q92551 via SWISS-MODEL. Five templates with superior GMQE (Global Model Quality Estimation) scores were employed for the model development, revealing that the AlphaFold DB model with a MolProbity score of 1.70 emerged as the most promising among them. Interestingly, this model surpassed others with MolProbity scores exceeding 2.0. However, the overall structural quality of the AlphaFold DB model required enhancement, as indicated by a clash score of 0.58, with its Ramachandran favored and Ramachandran outlier values estimated at 86.10% and 4.10%, respectively. To address this, the homology model underwent MD simulations for refinement, resulting in a final model with a significantly improved MolProbity score of 0.79. Crucially, this refinement led to the identification of 95.90% Ramachandran favored regions and no Ramachandran outliers, as depicted in the Supplementary Materials (Figure S1). This refined homology model was subsequently employed for molecular docking and MD simulations. 

**Figure 8:**
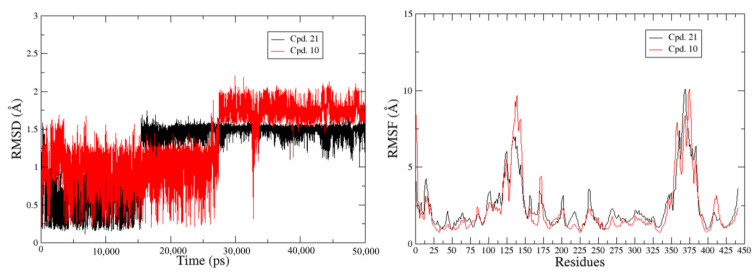
The ligand RMSD plots (**left**) and protein RMSF plots (**right**) for the higher-activity (**21**) and lower-activity (**10**) compounds.

Despite these improvements, a challenge persisted in identifying the ligands’ binding sites for IP6K1. To cope with this challenge, we utilized the CB-Dock2 web server for ligand binding site prediction, and the AutoDock Vina-based docking methodology was adopted to dock the input compounds into the three top-ranked cavities. Remarkably, both the most potent and least potent compounds were docked at the same binding cavity (located at *X* = 63 Å, *Y* = 77 Å, and *Z* = 40 Å, with a cavity volume extension of 801 Å^3^) and exhibited the maximum Vina scores. The docked poses of these compounds are shown in Figure S2 of the Supplementary Materials. These docked poses underwent 50 ns of explicit solvent MD simulations to unravel the dynamic behavior of the corresponding IP6K1 complexes. Our focus initially centered on the stability of the ligands at the proposed binding site. The ligand RMSD plots in Figure 8 reveal that the higher active compound **21** initially shifted from its binding pose but stabilized after 15 ns. In contrast, the lower active compound **10** also deviated from its initial binding pose, and although stabilization occurred just before 30 ns, it remained less stable compared to compound **21**. The RMSF plot indicates larger fluctuations in residues 125–180 for the lower-activity compound **10** compared to compound **21**. 

Subsequent MM-GBSA analyses were conducted to assess the enthalpic contribution of the binding free energy obtained from the MD simulations. The results in Table 6 indicate that compound **21** is predicted to have a significantly higher binding energy compared to compound **10**, primarily due to substantial variations in its electrostatic interactions. Compound **21** exhibited higher polar and non-polar interactions than compound **10**. The lower solvation free energy value obtained for compound **10**, possibly attributed to its smaller structure, contributed to an improved binding energy, but its lack of interactions with the amino acid residues reduced its overall binding energy.

**Figure 9:**
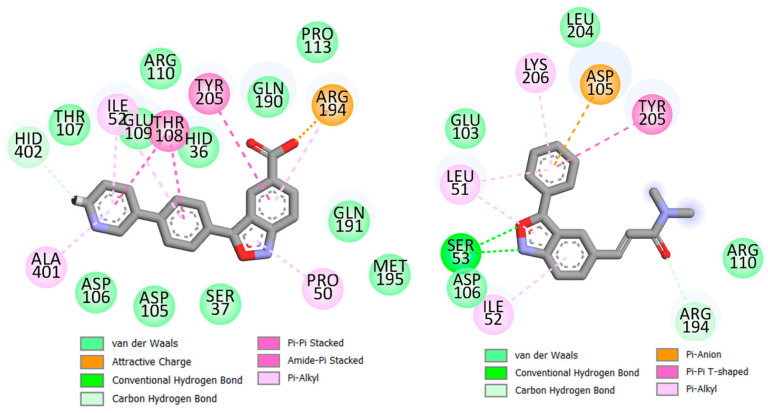
The final pose of compounds **21** (**left**) and **10** (**right**) within the IP6K1 receptor complexes obtained from the MD simulations.

In Figure 9, the average structures derived from the last 10 ns of the MD simulation are depicted. Naturally, compound **21** exhibits a greater number of interactions with the binding site amino acids compared to the less active compound **10**, with the majority of the additional interactions in the case of **21** being of a lipophilic nature. A robust polar interaction may be also observed between the carboxylate group of **21** and the Arg194 residue. Interestingly, this carboxylate formed strong polar interactions with both Gln190 and Arg194, playing a pivotal role in the stability of this ligand.

Conversely, the benzisoxazole moiety of the less active compound **10** formed hydrogen bond interactions with Ser53 and Asp106. The significance of these interactions was also underscored by the pharmacophore model. Consistent with the interpretations from the pharmacophore mapping, all three aromatic residues (including one phenyl and one benzisoxazole) of **21** engaged in π–π or π–amide interactions with Thr108 and Tyr205, while only one aromatic moiety of 10 formed such an interaction with Tyr205. Noticeably, the polar side chain of **10** explored the solvent environment and was less utilized in the interactions, whereas the hydrophobic residues of **21** engaged in a larger number of interactions with the amino acid residues. This information is reflected in the 2D-QSARs, where descriptors like CMC-50 and SpMax2_Bh(v) emerged as the most significant, as well as in the 3D-QSAR modeling, where the side chain of **10** was inserted into a steric unfavorable field.

The 3D-QSAR modeling particularly emphasized the significance of the pyridine moiety of **21**, as it is in close proximity to both electronegative and steric favorable fields. This ring was found to be involved in several interactions, including π–amide, π–alkyl, van der Waals, and carbon–hydrogen bonding. 

Understanding the role of hydrogen-bonding interactions is crucial given the distinct polar interactions that these ligands exhibit with different amino acids. To gain insights, trajectory analyses were carried out to estimate the hydrogen bond interactions. On average, it was observed that compound **21** forms a larger number of hydrogen bonds compared to compound **10**. Further analysis revealed that, for **21**, interactions between its carboxylate moiety and Arg194 and Gln190 are predominant. This specific interaction likely plays a pivotal role in providing substantial stability to **21** within the binding site. In contrast, for compound **10**, the major hydrogen bond interactions are those between the benzisoxazole moiety and Ser53.

**Figure 10:**
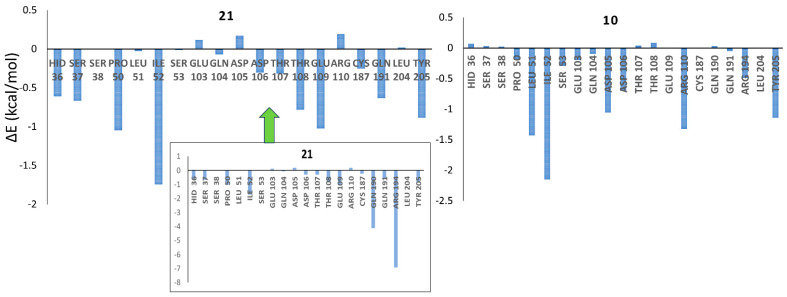
Total energy of the amino acid residues obtained from a per-residue decomposition analysis for complexes with compounds **21** and **10**.

In our pursuit of comprehensively understanding the contributions of different amino acid residues to the ligand binding of compounds **21** and **10**, we carried out a per-energy decomposition analysis. As shown in Figure 10, Arg194 and Gln190, which form hydrogen bond interactions with the carboxylate group of **21**, exhibited the maximum contributions to the binding of **21**. Notably, the interactions of **21** with these amino acid residues were 2–3 times higher than its interactions with other amino acid residues. The absence of such interactions in compound **10** substantiates the significant difference observed in the electrostatic energy (Δ*E*_elec_) values between **21** and **10**. To facilitate a clearer comparison of the other interactions between **21** and **10**, we have suppressed these two interactions in Figure 10. It is evident that interactions with amino acid residues such as His36, Ser37, Asp106, Thr107, Thr108, Glu109, and Glu191 were much more prominent for **21** compared to **10**. Additionally, most of these residues were found to engage in non-polar interactions with **21**. This later distinct analysis provides valuable insights into the distinct binding profiles of compounds **21** and **10** with the targeted amino acid residues.

## 3. Materials and Methods

### 3.1. Dataset Collection and Preparation 

Data on the structures of 36 IP6K1 inhibitors were collected from the recent study by Zhou et al. [26], in which a 50% inhibitory concentration (IC_50_) against IP6K1 was determined using an innovative enzyme-coupled assay technique. Subsequently, the IC_50_ values were transformed into pIC_50_ values, calculated as −log_10_(IC_50_/10^6^), and employed as response variables for setting up our ligand-based models. To ensure consistent data handling, the Simplified Molecular Input Line Entry System (SMILES) strings of these compounds, originally provided by Zhou et al., were then converted into canonical SMILES structures utilizing the RDKit tool and further translated into 3D .sdf format using the Discovery Studio Visualizer software. Further standardization of these 3D structures was performed using the Chemaxon Standardizer tool with the following steps: (a) the addition of explicit hydrogen atoms, (b) aromatization of the structures, (c) cleaning of both the 2D and 3D structures, (d) neutralization to ensure charge neutrality, and (e) salt stripping to eliminate any ionic compounds. Detailed information regarding the names, structures (SMILES notations), and pIC_50_ values of the dataset compounds is available in the Supplementary Materials (Table S1). The 2D structures of the dataset compounds are also shown in Figures S3 and S4 of the Supplementary Materials.

### 3.2. The 2D-QSAR Modeling 

#### 3.2.1. Descriptor Calculation 

The molecular descriptors were calculated using the alvaDesc v.2.0.4 module, accessible via the open-access OCHEM web platform (https://www.alvascience.com/alvadesc/) (accessed on 7 September 2023) [32]. The geometry of the 3D compound structures was optimized using the Corina tool in the OCHEM web server [33]. To create the final dataset for setting up the 2D-QSAR model, we combined the derived descriptors with the compounds’ pIC_50_ values.

#### 3.2.2. Dataset Division and Model Development

The entire dataset was split into a training set (80%) and a test set (20%) using the Python-coded open-access program SFS-QSAR-tool_v2 (https://github.com/ncordeirfcup/SFS-QSAR-tool, accessed on 12 September 2023) [34]. This dataset division was based on an activity sorting approach, starting with a value of 2. The division involved selecting every 5th compound as a training set, after sorting the values of pIC_50_ in descending order starting from the second compound. The 2D-QSAR models were then developed in two stages. Initially, we utilized descriptors from eight categories, offering high interpretability, including molecular characteristics, functional group counts, 2D atom pairs, drug-like indices, ring descriptors, atom-centered fragments, and constitutional descriptors. In the subsequent stage, we considered all categories of the alvaDesc descriptors for the model development. 

Regarding the 2D-QSAR modeling technique, we opted for a regression-based approach, specifically utilizing multiple linear regression (MLR) analysis. To build these MLR models, we employed two open-access software tools:

(a) (SFS-QSAR-tool_v2: This tool offers a graphical user interface for developing linear, interpretable *2*D-QSAR models. It uses the sequential forward selection (SFS) technique, which is based on the code available in the Mlxtend library (http://rasbt.github.io/mlxtend/, accessed on 12 September 2023). SFS is a non-stochastic feature selection strategy that resources various scoring functions and cross-validation strategies for selecting the most significant features for the 2D-QSAR models. In this work, four different scoring functions were employed, including the coefficient of determination (*R*^2^), the negative mean absolute error (NMAE), the negative mean Poisson deviance (NMPD), and the negative mean gamma deviance (NMGD). For each scoring function, models were generated both with no cross-validation and with 5-fold cross-validation, resulting in a total of eight (=4 × 2) models.
(b) Genetic-Algorithm v.4.1_2 (https://dtclab.webs.com/software-tools, accessed on 14 September 2023): This software generates linear interpretable MLR models using a stochastic genetic algorithm (GA) technique. The details of this methodology have been described elsewhere [35]. During the data processing, the correlation and variance cut-offs were set at 0.99 and 0.0001, respectively, to include a significant number of descriptors in the model development while excluding constant and highly correlated descriptors.

#### 3.2.3. Evaluation of the Models

To assess the quality of the generated 2D-QSAR models, we employed well-established validation parameters, namely *Q*^2^_LOO_ (Leave-One-Out cross-validated *R*^2^) and *R*^2^_Pred_*/Q*^2^_F1_ (predicted *R*^2^) [36,37]. While the former estimates the internal predictivity of the training set, the latter assesses the external predictivity of the test set. Since, for each set of descriptors, multiple models were generated using stochastic and non-stochastic feature selection strategies, these two parameters were crucial in selecting the most predictive 2D-QSAR model. 

Additional statistical parameters, such as *R*^2^, adjusted *R*^2^ (*R*^2^_adj_), mean absolute error (MAE), and *r_m_*^2^_LOO_, along with ∆*r_m_*^2^_LOO_ (for the training set), *r_m_*^2^_tes*t*_, ∆*r_m_*^2^_test_ (for the test set), *Q*^2^_F2_, and the root mean square error of prediction (RMSEP) [38], were also employed to evaluate the statistical quality of the final 2D-QSAR models. Moreover, the inter-collinearity among descriptors was assessed by inspecting the cross-correlation matrix, and the multicollinearity of the final models was examined using the variation inflation factor (VIF) [39]. The robustness of the models, based on the Y-randomization technique, was determined using the c*R*_p_^2^ value [40]. The applicability domain (AD) of the final models was estimated too using a Williams plot, which correlates standardized residuals (to identify response outliers) with leverages (to identify structural outliers) [38,41].

### 3.3. Ligand-Based Pharmacophore Modeling

To develop the structure-based pharmacophore models, we employed the recently introduced open-access tool, Quantitative Pharmacophore Activity Relationship (QPHAR) [42]. For each compound in the dataset, 50 conformations were generated using the genetic algorithm and Confab techniques separately, facilitated by the Open Babel software. 

The QPHAR models were built after dividing the dataset into a training set (26 compounds) and test set (10 compounds), using the splitData.py tool in QPHAR. The rationale behind the QPHAR-based pharmacophore modeling methodology has been described in detail by Kohlbacher et al. [42], as well as in our previous study [43]. Specifically, the train.py tool in this software was used for generating the models solely with the training set using the random forest (RF) technique and the following parameters: fuzzy: True; weight type: distance; threshold: 1.5; number of estimators: 10; maximum depth: 3; and metric: *R*^2^. The trained model was used for predicting the activity of the test set compounds by applying the predict.py tool. The internal predictivity of the derived pharmacophore models was evaluated by examining R^2^, the root mean square error (RMSE), the standard error (SE), and the median error (ME), while the external predictivity was simply assessed by calculating the *R*^2^_Pred_ value. 

The pharmacophore models aided in the structural alignment of the compounds. In this case, we used the profile3DActivity.py QPHAR application tool to obtain the pharmacophore-aligned structures, which were subsequently employed in the 3D-QSAR modeling [44].

### 3.4. The 3D-QSAR Modeling

In the 3D-QSAR modeling, the Open3DQSAR tool was employed with two different feature selection techniques: (a) Fractional Factorial Design-based variable SELection (FFD-SEL) and (b) Uninformative Variable Elimination-based Partial Least Square (UVE-PLS) [45,46]. 

To generate the models, we explored two different alignment techniques. First, we performed a simple unsupervised rigid body alignment method. For this, the input .sdf structures were geometrically optimized using the steepest descent technique under the MMFF94 force field. After minimization, 500 conformations of the ligand structures were generated using the rdMolAlign.GetCrippenO3A code in RDKit and subsequently employed for alignment. Notice that, in the current work, we generated 500 conformers instead of the usual 100 since the former yielded better models. A Python script, “alignment.py”, was written and used for the atom-based alignment, and it is available in the following GitHub repository: https://github.com/ncordeirfcup/InsilicoModeling_RdRp (accessed on 1 October 2023). 

The statistical predictivity of the models were assessed using parameters like *R*^2^; the *F*-test result; and leave-one-out (*Q*^2^_LOO_), leave-two-out (*Q*^2^_LTO_), and leave-many-out (*Q*^2^_LMO_ with 5 groups and 20 runs) cross-validation, as well as *R*^2^_Pred_. The contour maps were examined using isocontour values at partial least squares (PLS) coefficients of +0.002 (green) and −0.002 (yellow) for steric fields and of +0.001 (blue) and −0.001 (red) for electrostatic fields. A detailed description of the Open3DQSAR methodology can be found elsewhere [47].

### 3.5. Homology Modeling

To generate the homology model of IP6K1, we utilized the SWISS-MODEL web server with UniProt ID Q92551 (https://www.uniprot.org/, accessed on 8 October 2023) [48]. Following a template search and model development that employed multiple templates, we assessed the structural quality of the homology models using the MolProbity tool (http://molprobity.biochem.duke.edu/index.php, accessed on 10 October 2023) [49,50], accessible on the SWISS-MODEL web platform. This process led us to identify the AlphaFold protein structure (https://alphafold.ebi.ac.uk/, accessed on 10 October 2023) as producing the most reliable model [51,52]. Nevertheless, to further refine the AlphaFold model of IP6K1, we performed MD simulations using the Amber 20 software [53]. Specifically, we followed a step-by-step protocol for the model refinement as previously described by Nurisso et al. [54]. In summary, the protocol involved: (i) Minimization of the solvated (explicit) protein structure in two stages. The first stage included the minimization of the solvent and ions, followed by the second stage, in which the entire system was minimized; (ii) Heating the system in the NVT ensemble and conducting a 2 ns equilibration in the NPT ensemble; (iii) Carrying out a 50 ns explicit solvent MD simulation; and (iv) Minimizing the structure (without solvent or ions) under implicit solvent conditions. This involved running a total of 5000 cycles of conjugate gradient minimization under Generalized Born implicit solvation.

These steps were essential for ensuring the accuracy and reliability of the homology model of IP6K1, with the quality of the refined structure being further checked using MolProbity.

### 3.6. Molecular Docking Analysis

In this study, we employed a robust molecular docking strategy known as CB-Dock2, an upgraded version of CB-Dock recently introduced by Yang Cao and co-workers [55,56]. CB-Dock2, available at http://cao.labshare.cn/clab/index.html (accessed on 12 October 2023), offers several advantages, particularly in cavity searching using a protein-surface-curvature-based cavity detection approach called CurPocket. This approach aids in identifying potential binding sites within the protein. Indeed, CB-Dock2 was highly useful for both our homology models and when investigating unknown binding sites for the ligands. After identifying multiple cavities, we selected the top three CurPocket cavities based on the cavity volume for docking. We performed the docking using the software AutoDock Vina [57], as implemented in the CB-Dock2 web server. 

### 3.7. Molecular Dynamics Simulations

The docked complexes underwent extensive 50 ns MD simulations, following well-established procedures that have been described in detail elsewhere [58,59]. Briefly, the ligand parameterizations were conducted using Leap, implemented in Amber 14, employing the general AMBER force field (GAFF) in Antechamber. The MD simulations were carried out using the ff99SB force field with an explicit TIP3P water model in a cubic box, allowing for an 8 Å distance around the complexes. Moreover, both the Berendsen barostat and the Langevin thermostat were employed for keeping the pressure and temperature constant, respectively. To prepare the protein structures for simulation, we protonated them at a pH of 7.0 using the PDB2PQR web server (https://server.poissonboltzmann.org/pdb2pqr, accessed on 14 October 2023) [60]. Trajectory analyses were carried out using the PTRAJ and CPPTRAJ programs [61]; meanwhile, the results were visualized and plotted using the QtGrace software (https://sourceforge.net/projects/qtgrace/, accessed on 25 October 2023). Additionally, trajectory analyses enabled the determination of the hydrogen-bonding interactions between the ligands and the binding site amino acid residues of the FXR protein. The Molecular Mechanics Generalized Born Surface Area (MM-GBSA)-based enthalpic binding free energies of the ligands were estimated using the MMPBSA.py program in AMBER [62]. Furthermore, the entropy contribution (T∆S) was computed using normal mode analyses, with 100 snapshots collected from the final 10 ns. Normal mode analyses, which are based on a quasi-harmonic entropy approach, involve calculating the covariance matrix of the atomic displacements from their average positions across the sampled trajectory. Additionally, we assessed the energy contributions of the binding site amino acid residues using the per-residue free energy decomposition method and the Amber MM-GBSA module [58,62]. All the energy components, including the van der Waals, electrostatic, polar solvation, and non-polar solvation contributions, were calculated using 200 snapshots extracted from the last 10 ns of the MD run. These simulations provided critical insights into the ligand–receptor interactions and the stability of the complexes.

Finally, we evaluated the adsorption, distribution, metabolism, excretion, and toxicity (ADMET) profiles of the three most potent derivatives (i.e., compounds **21**, **15**, and **20**) using the admetSAR-2.0 web server (http://lmmd.ecust.edu.cn/admetsar2, accessed on 26 January 2024) [63]. The results are detailed in Table S2 of the Supplementary Materials. Due to their structural similarity, these compounds exhibited comparable ADMET profiles. All three compounds demonstrated low acute oral toxicity (category III: LD_50_ values greater than 500 mg/kg but less than 5000 mg/kg), moderate to poor water solubility, and favorable human oral bioavailability and intestinal absorption. Furthermore, the compounds displayed blood–brain barrier (BBB) permeability, as illustrated in the BOILED-Egg plot (depicted in Figure S5 of the Supplementary Materials), determined using the SwissADME web server (http://www.swissadme.ch/, accessed on 26 January 2024) [64]. Predictive analysis suggested the hepatotoxicity, reproductive toxicity, myopathy (OATP1B1 inhibitor), and respiratory toxicity of these derivatives. However, no signs of carcinogenicity, mutagenicity (Ames toxicity), cardiac toxicity (human ether-a-go-go-related gene inhibition), nephrotoxicity, or skin sensitization were observed. 

## 4. Conclusions

The pharmacological roles of IP6K1 have undergone extensive exploration in recent years, with emerging evidence suggesting that inhibitors of this kinase and its isoforms hold promise for the treatment of obesity and related metabolic disorders. Despite the importance of these findings, only a limited number of inhibitors targeting IP6K1 have been reported thus far. Consequently, this work represents the first comprehensive in silico ligand-based modeling effort focused on IP6K1 inhibitors. More importantly, the dataset used in this study exhibited more than three log unit differences in potency against the IP6K1 enzyme despite its limited structural diversity, rendering it particularly worthy of investigation.

The absence of a crystalline structure for human IP6K1 further underscores the relevance of ligand-based design for these inhibitors. The multifaceted objectives of this study began with the systematic development of validated and predictive ligand-based models. Among these models, the 2D-QSAR model developed using all alvaDesc descriptors demonstrated the highest statistical predictivity. Insights into the structural requirements of these compounds were also extracted from these models, emphasizing the balance between hydrophobic and electrostatic interactions, along with the 3D topology/geometry (such as CATS2D_01_LL [65], F04[C-C]), in ligand binding to the receptor. In particular, the best 2D-QSAR model found, developed with a limited number of descriptors, disclosed the structural and topological factors influencing higher or lower potencies. Additionally, the developed ligand-based pharmacophore model pinpointed the significance of aromatic ring, hydrophobic, and hydrogen bond acceptor features in determining the activity. The 3D-QSAR modeling, while providing limited information, clearly indicated how the electrostatic and steric requirements influence the biological activity against IP6K1. However, the homology model of IP6K1, along with subsequent molecular docking and MD simulations, revealed that polar interactions with residues like Glu190 and Arg194, particularly with the negative ionizable carboxylate group, significantly contribute to the potency against this kinase. In addition to these findings, hydrophobic, π–π, and π–amide interactions were identified as crucial to enhancing the stability of these compounds at the binding site. In conclusion, this work provides important guidelines for the design of novel IP6K1 inhibitors in the future. Furthermore, the most active compound in the dataset (i.e., compound **21**) exhibits a remarkable 26-fold selectivity toward IP6K1 and IP6K2 in comparison to IP6K3. Another compound in the dataset, compound **20**, demonstrates 4.2 times greater selectivity toward IP6K1 compared to IP6K3. Notably, compound **20** has shown efficacy in ameliorating obesity-related complications, including improvements in glycemic profiles, hepatic steatosis, and weight gain, without altering diet intake. This stands in contrast to other IP6K inhibitors such as SC-919 (IP6K1: IC_50_ < 5.6 nM; IP6K3: IC_50_ = 0.65 nM) and N2-(m-trifluorobenzyl)-N6-(p-nitrobenzyl)-purine (TNP) (IP6K1: IC_50_ = 270 nM; IP6K3 IC_50_ = 260 nM) [13,26]. These inhibitors hold promise for diverse pharmacological profiles, distinguishing them from the existing compounds, and warrant further exploration for their potential applications in obesity treatment. The generated ligand-based models can be employed for predicting the activity of novel derivatives, and the exploration of non-commercial software packages adds practical utility to the reported models. As a future avenue for this work, the docked complexes may undergo MD simulations with an extended time frame (e.g., 500 ns).

## Figures and Tables

**Table 1:** Summary of the statistical results obtained for the MLR models based on different types of descriptors ^a^.

Model ^a^	Score ^b^	CV ^c^	Interpretable Descriptors ^d^			Model^a^	All Descriptors		
			*Q* ^2^_LOO_	*R* ^2^_Pred_	Average		*Q* ^2^_LOO_	*R* ^2^_Pred_	Average
M01	*R* ^2^	none	0.733	0.427	0.580	M10	0.812	0.688	0.750
M02	NMAE	none	0.654	0.799	0.727	M11	0.820	0.868	0.844
M03	NMPD	none	0.733	0.427	0.580	M12	0.812	0.688	0.750
M04	NMGD	none	0.427	0.427	0.427	M13	0.829	0.405	0.617
M05	*R* ^2^	5	0.671	0.680	0.676	M14	0.704	0.526	0.615
M06	NMAE	5	0.662	0.253	0.458	**M15**	**0.839**	**0.870**	**0.855**
M07	NMPD	5	−0.898	0.605	−0.147	M16	0.823	0.849	0.836
M08	NMGD	5	−0.898	0.605	−0.147	M17	0.823	0.849	0.836
**M09**	GA-LDA	na	**0.800**	**0.785**	**0.793**	M18	0.840	0.801	0.821

^a^ The best models found are depicted in bold. ^b^ Scoring function used in SFS-based feature selection. ^c^ Cross-validation used in the SFS-based feature selection. ^d^ Descriptors belonging to the eight specific categories, as outlined in the Materials and Methods section (Section 3.).

**Table 2:** Statistical results for the best 2D-QSAR models found, M09 and M15.

Equation	Statistical Results
**Model M09 (Interpretable descriptors)** pIC_50_ = +0.169(±0.028) F04[C-C] −0.447(±0.139) CMC-50 −0.378(±0.131) nRCONHR −0.275(±0.029) H-047 +0.1(±0.031) CATS2D_01_LL + 5.125(±0.485)	*N*_training_ = 29, *R*^2^ = 0.857, *R*^2^_adj_ = 0.826, *Q*^2^_LOO_ = 0.800, MAE = 0.201, *r*_m_^2^_LOO_ = 0.724, ∆*r*_m_^2^_LOO_ = 0.088 *N*_test_ = 7, *R*^2^_Pred_/*Q*^2^_F1_ = 0.785, *Q*^2^_F2_ = 0.765, RMSEP = 0.309, *r*_m_^2^_test_ = 0.706, ∆*r*_m_^2^_test_ = 0.125
**Model M15 (All descriptors)** pIC_50_ = +0.223(±0.083) VE3sign_B(s) +3.079(±0.574) MATS4m +10.797(±1.593) SpMax2_Bh(v) −6.694(±1.071) G3i +10.984(±3.07) R5e+ −33.064(±6.179)	*N*_training_ = 29, *R*^2^ = 0.890, *R*^2^_adj_ = 0.866, *Q*^2^_LOO_ = 0.839, MAE = 0.181, *r_m_*^2^_LOO_ = 0.772, ∆*r_m_*^2^_LOO_ = 0.117 *N*_test_ = 7, *R*^2^_Pred_/*Q*^2^_F1_ = 0.870, *Q*^2^_F2_ = 0.858, RMSEP = 0.240, *r_m_*^2^_test_ = 0.740, ∆*r_m_*^2^_test_ = 0.120

**Table 3:** The five descriptors appearing in the 2D-QSAR model M15.

Descriptor	Definition	Category
R5e+	R maximal autocorrelation of lag 5 weighted by Sanderson electronegativity	GETAWAY
SpMax2_Bh(v)	Largest eigenvalue n. 2 of Burden matrix weighted by van der Waals volume	Burden eigenvalues
G3i	Third-component symmetry directional WHIM index weighted by ionization potential	WHIM
MATS4m	Moran autocorrelation of lag 4 weighted by mass	2D autocorrelations
VE3sign_B(s)	Logarithmic coefficient sum of the last eigenvector from Burden matrix weighted by I-State	2D matrix-based

**Table 4:** Statistical results for the best QPHAR-based pharmacophore model found.

Parameter	Training	Test
*N*	26	10
*R* ^2^	0.845	
RMSE	0.309	
ME	0.248	
SE	0.183	
*R* ^2^_Pred_		0.565
*R* ^2^_Pred_^a^		0.716

^a^ After removal of one outlier.

**Table 5:** Statistical results of 3D-QSAR models using different feature selection techniques ^a^.

Parameter ^b^	FFD-SEL	UVE-PLS
*N*_training_	29	29
*NC* ^b^	4	3
*R*^2^ (SDEC)	0.912 (0.176)	0.856 (0.224)
*F*	62.157	33.847
*Q*^2^_LOO_ (SDEP)	0.637 (0.357)	0.370 (0.471)
*Q*^2^_LTO_ (SDEP)	0.626 (0.363)	0.361 (0.474)
*Q*^2^_LMO_ (SDEP)	0.573 (0.387)	0.311 (0.492)
*N*_test_	7	7
*R*^2^_Pred_ (SDEP)	0.747 (0.564)	0.668 (0.646)
*Q* ^2^ *_s_*	0.428	---

^a^ FFD-SEL: Fractional Factorial Design-based variable SELection; UVE-PLS: Uninformative Variable Elimination-based Partial Least Square. ^b^ *NC*: Number of principal components; SDEC: Standard error of calculation; SDEP: Standard error of prediction.

**Table 6:** Calculated binding free energies [Δ*G*_bind_(T)] for selected IP6K1 complexes ^a^. All the components shown are in kcal/mol.

Complexes	Δ*E*_vdW_	Δ*E*_elec_	Δ*G*_gas_	Δ*G*_polar_	Δ*G*_non-polar_	Δ*G*_solvation_	T∆*S*	Δ*G*_bind_(T)
**21**	−42.45	−125.22	−167.67	+133.91	−5.78	+128.13	−21.20	−18.35
**10**	−38.26	−5.73	−43.99	+18.32	−4.25	+14.07	−24.26	−5.67

^a^ Δ*G*_bind_(T): theoretical binding free energy (Δ*G*_bind_(T) = Δ*E*_vdW_ + Δ*E*_elec_ + Δ*G*_polar_ + Δ*G*_non-polar_ − TΔ*S*) and its components, namely Δ*E*_vdW_: van der Waals interaction energy; Δ*E*_elec_: electrostatic interaction energy; Δ*G*_polar_: polar solvation free energy; Δ*G*_non-polar_: non-polar solvation free energy, TΔ*S*: entropy.

## Data Availability

The dataset used in this work is provided in the Supplementary Materials. The other data associated with this work (the results of the models) will be made available on request.

## Supplementary Materials

The following supporting information can be downloaded at https://www.mdpi.com/article/10.3390/ph17020263/s1. Figure S1: Ramachandran plot of the generated homology model for IP6K1; Figure S2: The docked poses of (A) the most active (**21**) and (B) the least active compound (**10**); Figure S3: Two-dimensional structures of the dataset compounds (**3**–**24**); Figure S4: Two-dimensional structures of the dataset compounds (**25**–**42**); Figure S5: The BOILED-Egg plot of three most potent compounds of the datasets (**15, 20**, and **21**); Table S1: Serial numbers, structures, and biological activities of the dataset compounds; Table S2. The ADMET properties of three most potent compounds of the datasets (**15**, **20**, and **21**).
