# Supplementary material for: Shaping the Future of Obesity Treatment: In Silico Multi-Modeling of IP6K1 Inhibitors for Obesity and Metabolic Dysfunction
Source: Pharmaceuticals (Basel). 2024 Feb 19;17(2):263. doi: 10.3390/ph17020263 (PMC10891520; doi:10.3390/ph17020263)
Supplement: Supplementary file 1 [file pharmaceuticals-17-00263-s001.zip › Supplementary_data.pdf]

Shaping the Future of Obesity Treatment: *In Silico* Multi-Modeling of IP6K1 Inhibitors for Obesity and Metabolic Dysfunction

Ismail Mondal[1], Nirupam Pattanayak[1], Sudip Kumar Mandal[1], Amit Kumar Halder[1,2], M. Natalia D. S. Cordeiro[2]*

[1]Dr. B. C. Roy College of Pharmacy and Allied Health Sciences, Dr. Meghnad Saha Sarani, Bidhannagar, Durgapur 713206, West Bengal, India
[2]LAQV@REQUIMTE/Department of Chemistry and Biochemistry, Faculty of Sciences, University of Porto, 4169-007 Porto, Portugal.

* **Corresponding author**: M. Natalia D. S. Cordeiro, ncordeir@fc.up.pt

Associated content:

Figure S1. Ramachandran plot of the generated homology model for IP6K1.

Figure S2. The docked poses of (A) the most active (**21**) and (B) the least active compound (**10**).

Figure S3. 2D structures of the dataset compounds (**3-24**)

Figure S4. 2D structures of the dataset compounds (**25-42**)

Figure S5. The Boiled-egg plot of three most potent compounds of the datasets (**15**, **20** and **21**)

Table S2. The ADMET properties of three most potent compounds of the datasets (**15**, **20** and **21**)

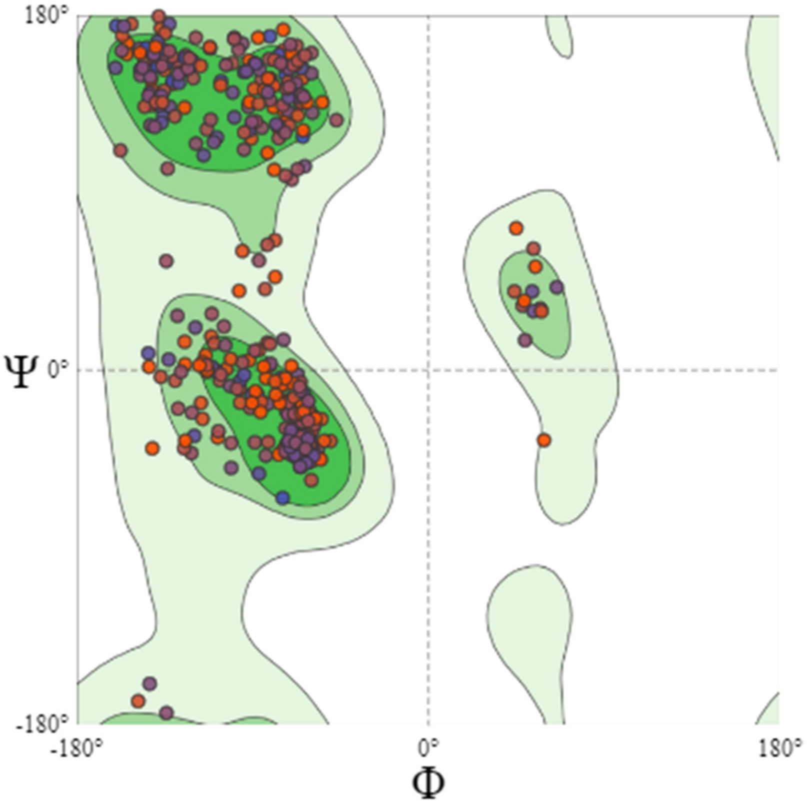

Figure S1. Ramachandran plot of the generated homology model for IP6K1.

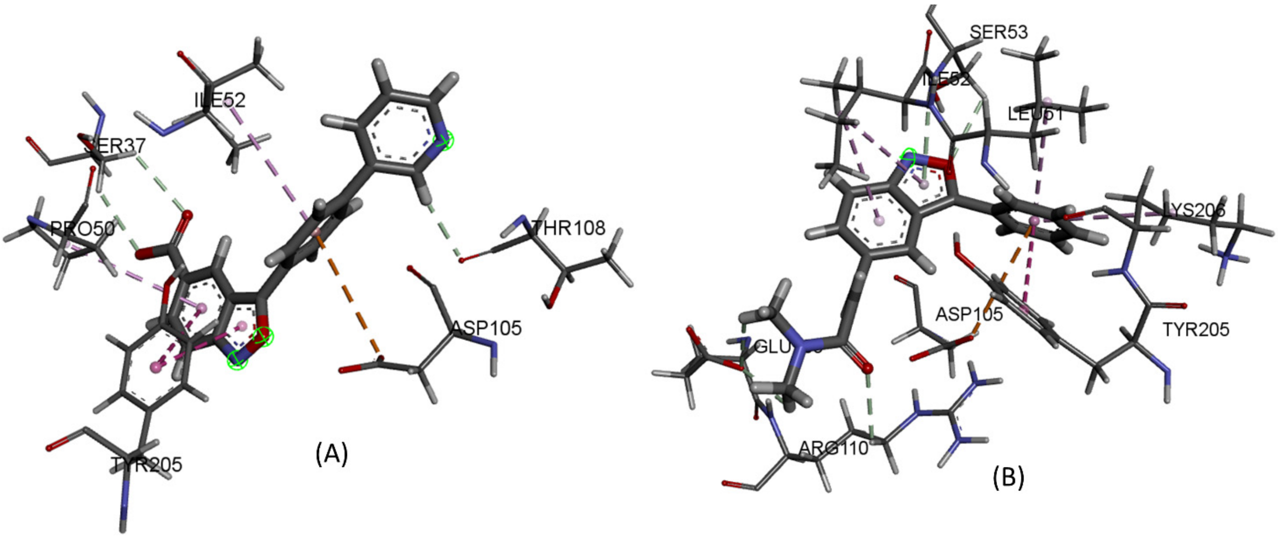

Figure S2. The docked poses of (A) the most active (**21**) and (B) the least active compound (**10**)

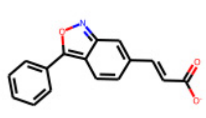

3, pIC50: 5.523

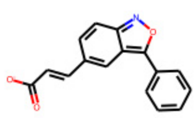

6, pIC50: 6.066

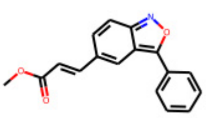

9, pIC50: 5.25

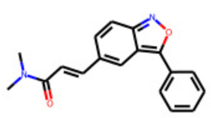

10, pIC50: 4.53

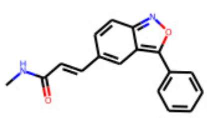

11, pIC50: 4.693

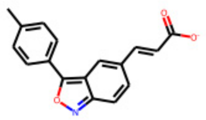

12, pIC50: 5.807

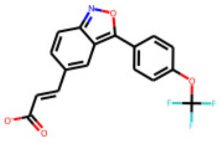

13, pIC50: 6.886

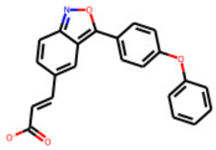

14, pIC50: 6.886

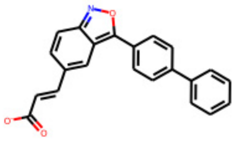

15, pIC50: 7.569

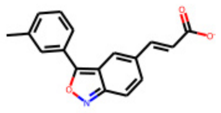

16, pIC50: 6.796

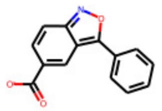

17, pIC50: 6.041

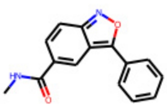

18, pIC50: 5.504

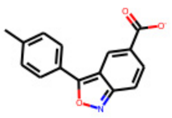

19, pIC50: 5.951

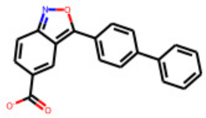

20, pIC50: 7.244

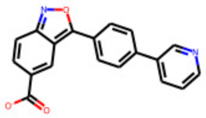

21, pIC50: 7.585

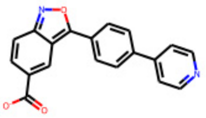

22, pIC50: 6.77

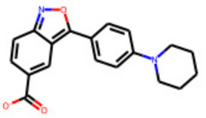

23, pIC50: 6.62

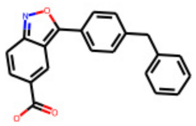

24, pIC50: 6.959

Figure S3. 2D structures of the dataset compounds (**3-24**)

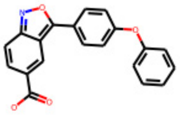

25, pIC50: 6.347

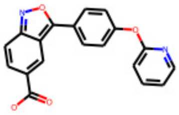

26, pIC50: 5.36

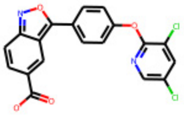

27, pIC50: 6.367

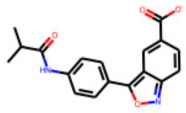

28, pIC50: 6.367

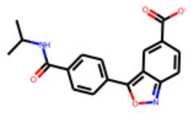

29, pIC50: 6.432

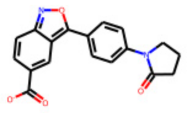

30, pIC50: 6.495

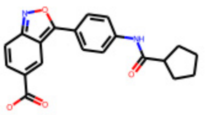

31, pIC50: 6.721

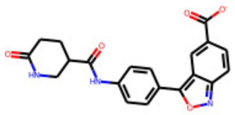

32, pIC50: 5.728

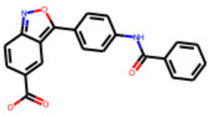

33, pIC50: 6.149

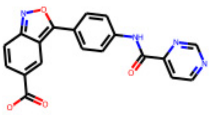

34, pIC50: 6.097

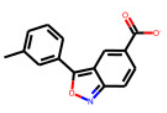

35, pIC50: 6.553

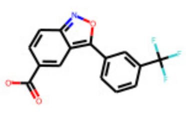

36, pIC50: 5.807

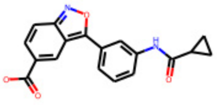

37, pIC50: 6.155

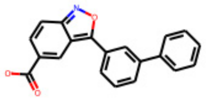

38, pIC50: 7.092

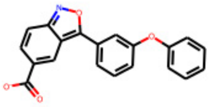

39, pIC50: 6.77

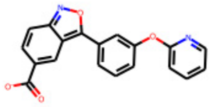

40, pIC50: 6.215

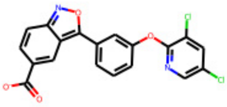

41, pIC50: 7.108

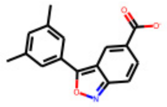

42, pIC50: 7.076

Figure S4. 2D structures of the dataset compounds (**25-42**)

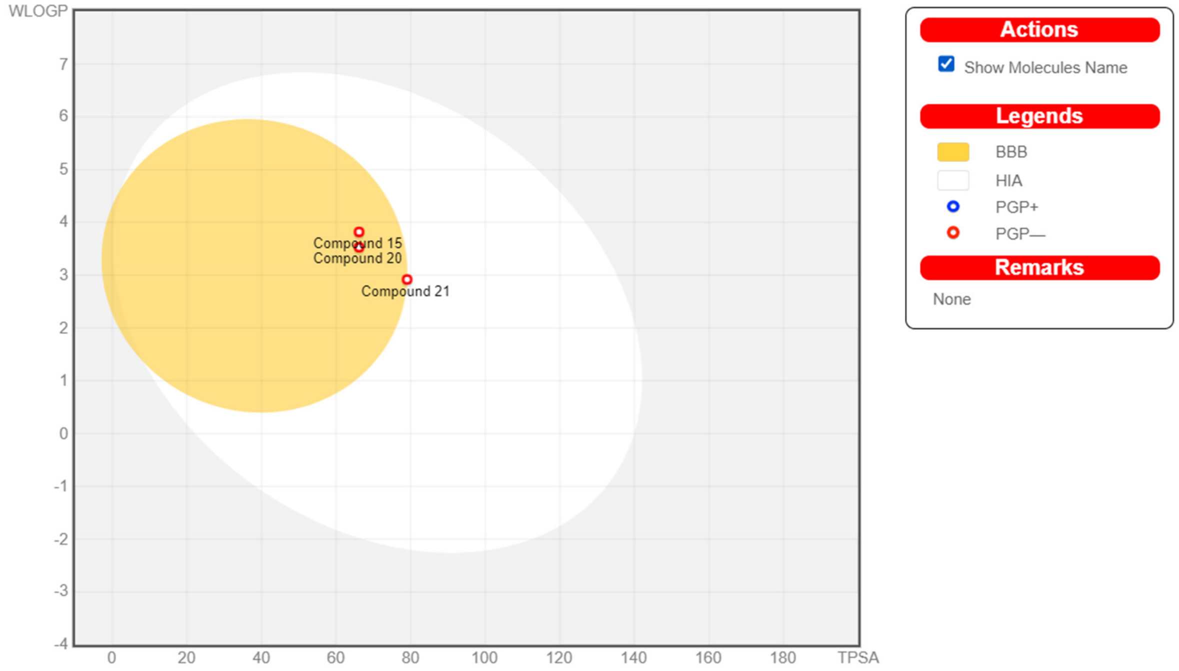

Figure S5. The Boiled-egg plot of three most potent compounds of the datasets (**15**, **20** and **21**)

Table S2. The ADMET properties of three most potent compounds of the datasets (**15**, **20** and **21**)

ADMET property	Compound 21	Compound 15	Compound 20
Ames mutagenesis	-	-	-
Acute Oral Toxicity (c)	III	III	III
Androgen receptor binding	+	+	+
Aromatase binding	+	+	+
Blood Brain Barrier	+	+	+
BRCP inhibitior	-	-	+
Biodegradation	-	-	-
BSEP inhibitior	+	+	+
Caco-2	-	-	-
Carcinogenicity (binary)	-	-	-
CYP1A2 inhibition	+	+	+
CYP2C19 inhibition	-	+	-

CYP2C8 inhibition	+	+	+
CYP2C9 inhibition	-	-	-
CYP2C9 substrate	-	-	-
CYP2D6 inhibition	-	-	-
CYP2D6 substrate	-	-	-
CYP3A4 inhibition	-	-	-
CYP3A4 substrate	-	-	-
CYP inhibitory promiscuity	-	+	-
Eye corrosion	-	-	-
Eye irritation	-	+	-
Estrogen receptor binding	+	+	+
Glucocorticoid receptor binding	+	+	+
Hepatotoxicity	+	+	+
Human Ether-a-go-go-Related Gene inhibition	-	+	-
Human Intestinal Absorption	+	+	+
Human oral bioavailability	+	+	+
MATE1 inhibitor	-	-	-
Mitochondrial toxicity	-	-	-
Micronuclear	+	+	+
Nephrotoxicity	-	-	+
Acute Oral Toxicity	2.382	1.712	2.283
OATP1B1 inhibitor	+	+	+
OATP1B3 inhibitor	+	+	+
OATP2B1 inhibitor	-	-	-
OCT1 inhibitor	-	-	-
OCT2 inhibitor	-	-	-
P-glycoprotein inhibitior	-	-	-
P-glycoprotein substrate	-	-	-
PPAR gamma	+	+	+
Plasma protein binding	0.895	0.961	1.001
Reproductive toxicity	+	+	+
Respiratory toxicity	+	+	+
Skin corrosion	-	-	-
Skin irritation	-	-	-
skin sensitisation	-	-	-
Subcellular localisation	Mitochondria	Plasma membrane	Mitochondria
Thyroid receptor binding	+	+	+
UGT catalysed	-	-	-
Water solubility	-3.862	-4.144	-3.864
